# Genome-wide identification of direct HBx genomic targets

**DOI:** 10.1186/s12864-017-3561-5

**Published:** 2017-02-17

**Authors:** Francesca Guerrieri, Laura Belloni, Daniel D’Andrea, Natalia Pediconi, Loredana Le Pera, Barbara Testoni, Cecilia Scisciani, Oceane Floriot, Fabien Zoulim, Anna Tramontano, Massimo Levrero

**Affiliations:** 1 Center for Life NanoScience@Sapienza, Istituto Italiano di Tecnologia, Viale Regina Elena 291, Rome, 00161 Italy; 2 Biocomputing Lab, Department of Physics, Sapienza University, Rome, Italy; 3 Department of Molecular Medicine, Sapienza University, Viale Regina Elena 291, Rome, 00161 Italy; 4 INSERM U1052, Cancer Research Center of Lyon (CRCL), 151 cours Albert Thomas, Lyon, 69424 France; 5 Department of Internal Medicine - DMISM, Sapienza University, Viale del Policlinico 155, 00161 Rome, Italy; 6 Istituto Pasteur Fondazione Cenci Bolognetti, Viale Regina Elena 291, Rome, 00161 Italy; 7 Cancer Research Center of Lyon (CRCL) - INSERM U1052, 151 cours Albert Thomas, 69424 Lyon Cedex 03, France

**Keywords:** Hepatitis B virus, HBx, Epigenetics, miRNAs, ChIP-Seq

## Abstract

**Background:**

The Hepatitis B Virus (HBV) HBx regulatory protein is required for HBV replication and involved in HBV-related carcinogenesis. HBx interacts with chromatin modifying enzymes and transcription factors to modulate histone post-translational modifications and to regulate viral cccDNA transcription and cellular gene expression. Aiming to identify genes and non-coding RNAs (ncRNAs) directly targeted by HBx, we performed a chromatin immunoprecipitation sequencing (ChIP-Seq) to analyse HBV recruitment on host cell chromatin in cells replicating HBV.

**Results:**

ChIP-Seq high throughput sequencing of HBx-bound fragments was used to obtain a high-resolution, unbiased, mapping of HBx binding sites across the genome in HBV replicating cells. Protein-coding genes and ncRNAs involved in cell metabolism, chromatin dynamics and cancer were enriched among HBx targets together with genes/ncRNAs known to modulate HBV replication. The direct transcriptional activation of genes/miRNAs that potentiate endocytosis (Ras-related in brain (RAB) GTPase family) and autophagy (autophagy related (ATG) genes, beclin-1, miR-33a) and the transcriptional repression of microRNAs (miR-138, miR-224, miR-576, miR-596) that directly target the HBV pgRNA and would inhibit HBV replication, contribute to HBx-mediated increase of HBV replication.

**Conclusions:**

Our ChIP-Seq analysis of HBx genome wide chromatin recruitment defined the repertoire of genes and ncRNAs directly targeted by HBx and led to the identification of new mechanisms by which HBx positively regulates cccDNA transcription and HBV replication.

**Electronic supplementary material:**

The online version of this article (doi:10.1186/s12864-017-3561-5) contains supplementary material, which is available to authorized users.

## Background

Despite the availability of an effective prophylactic vaccine and potent antiviral therapies hepatitis B virus (HBV) is still a major health problem. Over 240 million people chronic hepatitis B virus (HBV) carriers worldwide remain at risk of developing hepatocellular carcinoma (HCC) [1, 2]. The persistence of viral replication in the liver and high serum HBV-DNA levels correlate with disease severity, progression of liver fibrosis and HCC development in the clinical setting [2].

The HBV regulatory protein HBx is both required for HBV replication [3] and implicated in HBV-related oncogenesis [4]. The mechanisms underlying the pleiotropic activities of HBx have been only partially elucidated. HBx regulates transcription both directly, at the chromatin level, and indirectly, by affecting intracellular signaling pathways that modulate the activity of multiple transcription factors [4]. HBx has been shown to target the epigenetic control of cellular genes expression by interacting with chromatin modifying enzymes [5–7]. HBx is also recruited to the cccDNA in HBV-infected cells [3] and is required for the transcription of all viral RNAs from the cccDNA minichromosome in the nucleus [3, 8]. HBx is thought to regulate cccDNA transcription by two main mechanisms: a) the degradation of the Smc5/6 restriction factor mediated by the DDB1 – Cul4 E3 ligase complex [9, 10] and b) the prevention of cccDNA epigenetic silencing by the histone deacetilase HDAC1 [8], the protein arginine N-methyltransferase 1 (PRMT1) [11], the Tudor-domain protein Spindlin-1 [12] and the histone methyl-transferase SETDB1 [13, 14]. Additional HBx activities that boost HBV replication are DNA Methyltransferase 3 Alpha (DNMT3A) downregulation mediated by miR-101 induction [15], the elevation of cytosolic calcium levels [16] and the induction of autophagy [17].

## Results

### ChIP-Seq analysis of genome wide HBx recruitment

To obtain a high-resolution, unbiased, mapping of HBx binding sites across the genome we sequenced HBx-bound fragments by ChIP-Seq in a cccDNA-driven HBV replication system [18]. Four independent anti-HBx paired ChIPs were carried out on formaldehyde-crosslinked chromatin from mock and HBV-transfected HepG2 cells. On average, ~62% of the reads could be aligned to the hg19 version of the human genome (Additional file 1: Figure S1a); ~1% of the total number of reads aligned to the HBV genome (ayw strain, NCBI Reference Sequence: NC_003977.2) and ~1% of reads falls on the mitochondrial genome. All reads with multiple alignments and mismatches with the reference genomes were excluded (~6.3% with at least one unknown base, ~11% with multiple alignment and ~13% with 1 or 2 mismatches). ~4000 statistically significant peaks/run (*P*-value < 10^−4^) were detected. About 12% of the HBx peaks were located within 10kb from the transcription start site (TSS) of known genes, 44.6% were located in intergenic regions and 39.5% within introns (Additional file 1: Figure S1b-S1c).

### Functional analysis of the genes potentially regulated by HBx

**Fig. 1:**
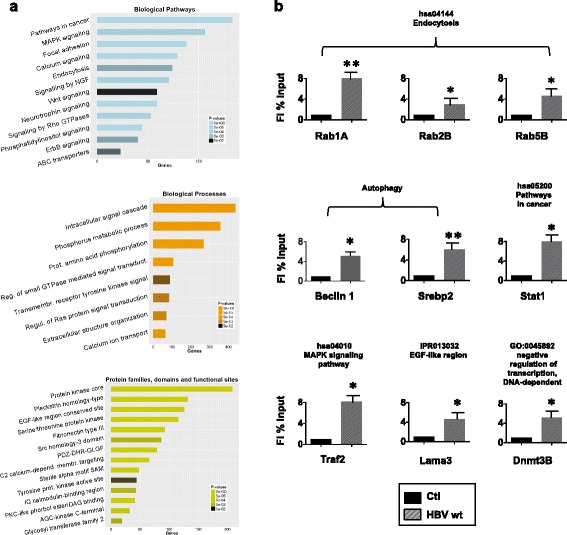
Functional analysis of protein-coding genes potentially regulated by HBx. **a** Biological Pathways (KEGG, REACTOME), Biological Processes (GO) and Protein families, domains and important sites (InterPro) over-represented (Benjamini-corrected *p*-value < 5×10^−2^) from the analysis of ~5500 protein-coding genes with HBx-binding sites are shown. In each plot, the categories are in descending order with respect to the associated gene number, with the corresponding color scale of p-values shown on the *right*. **b** Validation of HBx occupancy on selected gene promoters in independent ChIP experiments. ChIP results are expressed as Fold Induction (FI) of the % of Input. Histograms show the mean from three independent experiments; *bars* indicate SD. P-values: * 0,02 ≤ *P* < 0,05; ** 0,01 ≤ *P* < 0.02; *** 0,005 ≤ *P* < 0.01. Gene specific promoter primer pairs are detailed in Additional file 1: Table S6

We first performed a functional enrichment analysis of the whole genome repertoire of the genes potentially regulated by HBx in HBV replicating HepG2 cells. Figure 1a shows the categories enriched, respectively, among the *KEGG* and *Reactome* Biological Pathways; the *Gene Ontology* (GO) Biological Processes and the *InterPro families*, a database that integrates diverse information about protein families, domains and functional sites. Overall, the results show a significant enrichment in genes involved in cell metabolism, chromatin dynamics and cancer, but also in biological pathways that have been associated with the control of HBV replication [i.e. Ras/Src [19], calcium transport, endocytosis]. It is important to underline that, since the HBx ChIP-Seq dataset was generated in HBV replicating HepG2 cells, which are derived from a primary liver tumor, and that an enrichment in genes belonging to the metabolism, chromatin dynamics and cancer pathways is often found in many immortalized and transformed cell lines, a number of the HBx genomic binding sites we found might reflect a bias for transcriptionally active chromatin regions in the “tumor cell” chromatin environment.

Independent anti-HBx ChIP experiments confirmed HBx recruitment, with a 3 to 8-fold enrichment relative to the control IgG ChIPed samples, to the promoter regions of selected genes belonging to different functional categories [Hsa04144: Rab1A, Rab2B, Rab5B; GO:0006357: SREBP2, Beclin1; Has04010: Traf2; Hsa05200: Stat1; IPR013032: Lama3; GO:0045892: Dnmt3B] in HBV replicating HepG2 cells (Fig. 1b) and in HBV-infected NTCP-HepG2 cells (Additional file 1: Figure S2a) and primary human hepatocytes from 2 different donors (Additional file 1: Figure S2b).

Validated PCR primers for genomic target sites that were negative for anti-HBx ChIP-Seq peak assignment showed no enrichment in the HBx immunoprecipitated chromatin, thus confirming the lack of off-target HBx recruitment and the specificity of the anti-HBx ChIP (Additional file 1: Figure S3).

### HBx directly enhances endocytosis

**Fig. 2:**
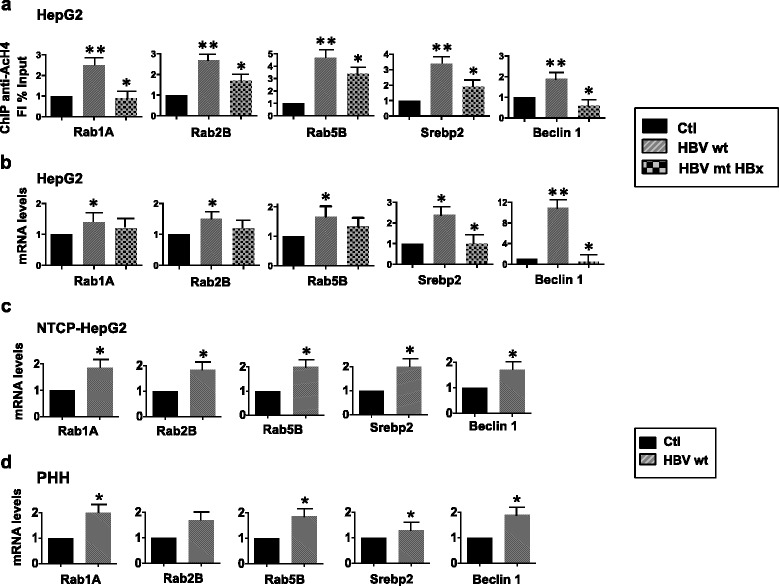
HBx ChIP-seq identifies direct target genes involved in endocytosis. **a** H4 acetylation on Rab1A, Rab2B, Rab5B, Srebp2 and Beclin 1 genes promoters assessed in independent ChIP experiments. Anti-AcH4 ChIPed chromatin from mock, HBV-wt and HBx-mt HBV replicating HepG2 cells and NTCP-HepG2 HBV-infected cells. Results are expressed as in Fig. 1b. **b**–**d** qPCR quantification of Rab1A, Rab2B Rab5B, Srebp2 and Beclin-1 mRNA levels in mock, HBV-wt and HBx-mt HepG2 cells replicating HBV (**b**) HBV-infected NTCP-HepG2 (**c**) and PHHs (**d**). Results are expressed as fold induction relative to the mock-transfected/infected controls (Ctl) after normalization to endogenous human β-actin mRNAs. Data represent means ± SD from at least three independent experiments performed in duplicate. P-values: * 0,02 ≤ *P* < 0,05; ** 0,01 ≤ *P* < 0.02; *** 0,005 ≤ *P* < 0.01

**Fig. 3:**
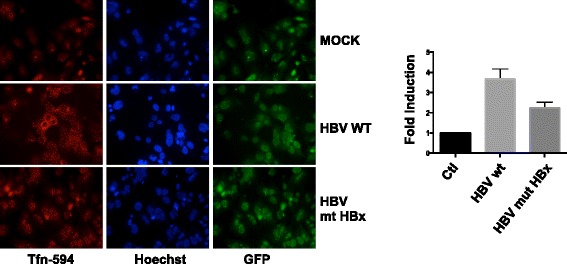
HBx directly enhances endocytosis. Endocytosis of transferrin is increased in HBV-wt replicating HepG2 cells. Representative images of Alexa Fluor 594-conjugated human transferrin uptake (*red*, *left panels*), nuclear staining with Hoechst (*blue*, *middle panels*) and nuclear GFP positive transfected cells (*green*, *right panels*) in mock, HBV-wt and HBx-mt HBV transduced HepG2 cells. Histograms represent the mean and SD of Alexa 488-conjugated transferrin signal quantified by ImageJ software in 100 cells from each of 3 independent experiments

The enrichment of genes involved in endocytosis among HBx targets (Fig. 1a) represents a potential link between its role in viral replication [20, 21] and its contribution to liver cancer development [22]. Our ChIP-Seq analysis showed that HBx is recruited and potentially regulates 24 members of the Ras-related in brain (RAB) GTPase family of genes, including several RAB GTPases implicated in endocytosis (Additional file 1: Table S1). HBx binding to the RAB1A, RAB2B and RAB5B promoter regions (see Fig. 1b) was accompanied by an increase in promoter-bound histone H4 acetylation (Fig. 2a) and RAB1A, RAB2B and RAB5B expression (Fig. 2b) in HBV-wt replicating cells but not in HBx-mt replicating cells. The upregulation of RAB1A, RAB2B and RAB5B expression was confirmed in HBV-infected NTCP-HepG2 cells (Fig. 2c) and human primary hepatocytes (PHH) (Fig. 2d). The specificity of HBx binding to the RAB1A, RAB2B and RAB5B promoter regions was further confirmed in NTCP-HepG2 cells infected with HBV-wt and HBx-mt virus (Additional file 1: Figure S4). Notably, HBV-wt HepG2 replicating cells showed a robust uptake of transferrin, with intense perinuclear accumulation, whereas transferrin distribution was more diffuse in mock HepG2 cells and in HBx-mt HepG2 replicating cells due to a diminished rate of transferrin internalization (Fig. 3).

HBx is also recruited to and potentially regulates several genes involved in autophagy, including Beclin-1, SREBP2 and several members of the ATG (AuTophaGy related) gene family (Additional file 1: Table S1). HBV has been show to induce ER stress and cellular autophagy, which is known to enhance viral replication and virions assembly [17]. Beclin-1 is a major effector of autophagy in mammalian cells and has been shown to be upregulated in HBV-related HCCs, HBx-overexpressing cells and 2.2.15 HBV replicating cells [23]. The SREBP-2 transcription factor, known to be a major regulator of cholesterol metabolism, has also been involved in the direct activation of several genes involved in autophagy [24]. HBx recruitment to the Beclin-1 and SREBP2/miR-33a promoter regions was confirmed in independent ChIP experiments performed in HBV-wt replicating cells (Fig. 1b). HBV-wt replicating cells, but not HBx-mt replicating cells, displayed higher levels of histone H4 acetylation on the Beclin-1 and SREBP2 promoters as compared to HBV mock cells (Fig. 2a) and increased expression of Beclin-1 and SREBP2 (Fig. 2b). These results were confirmed in HBV-infected NTCP-HepG2 cells (Fig. 2c) and HBV-infected human primary hepatocytes (PHH) (Fig. 2d). Altogether, these results indicate that HBx targets endocytosis and autophagy gene expression in HBV replicating cells.

### ChIP-Seq identifies ncRNAs directly targeted by HBx

**Fig. 4:**
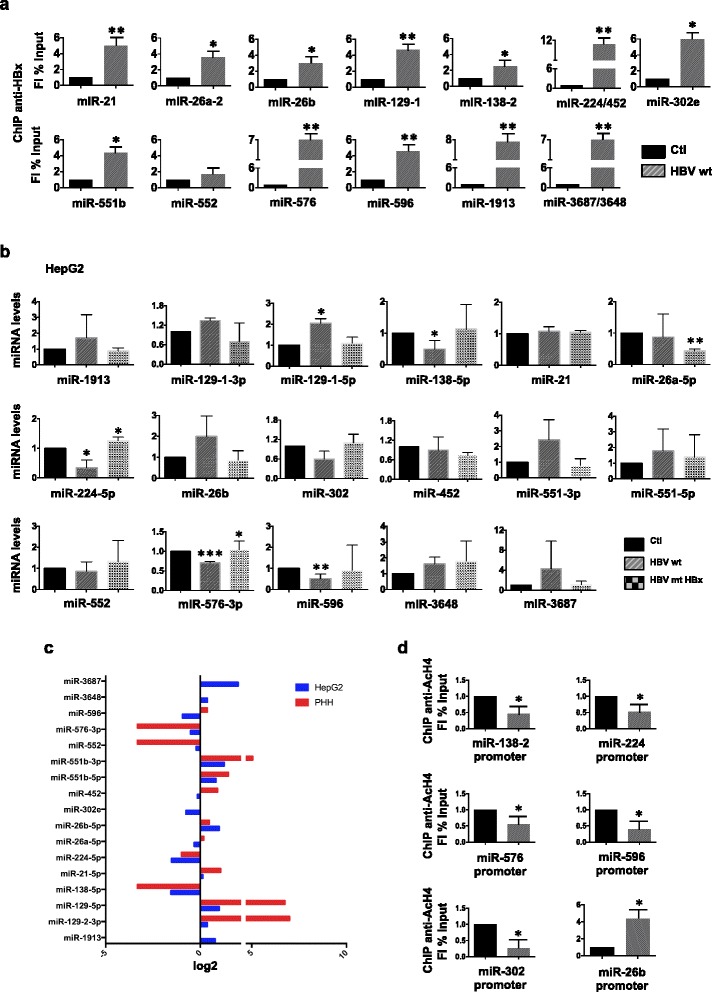
ChIP-seq miRNA peak validation and HBx targeted miRNAs expression. **a** HBx occupancy on the putative promoter regions (−5000 + 1000 nt relative to position +1 of each miRNA in MirBase v18) of selected miRNAs (Additional file 1: Figure S1d) was validated in independent ChIP experiments. Cross-linked chromatin from mock or HBV-wt replicating HepG2 cells was immunoprecipitated with a specific anti-HBx antibody or relevant IgG controls, and then analyzed by real-time qPCR using specific primer pairs (see Additional file 1: Table S6). ChIP results are expressed as Fold Induction (FI) of the % of Input and the histograms show the mean from three independent experiments; *bars* indicate SD. **b**–**c** miRNA profiles were analyzed by real-time qPCR, and normalized with respect to RNU38, in mock, HBV-wt and HBx-mt replicating HepG2 cells (**b**) and by Taqman PCR-arrays in HBV infected (12 dpi) PHHs (**c**). Data represent means ± SD from at least three independent experiments performed in duplicate. **d** HBx recruitment impacts on H4 histone acetylation of neighboring chromatin promoters. Anti-AcH4 ChIPs were performed and analysed as in *a*). P-values: * 0,02 ≤ *P* < 0,05; ** 0,01 ≤ *P* < 0.02; *** 0,005 ≤ *P* < 0.01

Analyses of the distribution of all the anti-HBx ChIP-Seq peaks revealed the occurrence of putative HBx binding sites in 16 lncRNA promoters and 32 lncRNA intragenic regions, in 44 snoRNA, 3 snRNA and 75 miRNA promoter regions (Additional file 1: Figure S1d). 39 out of the 75 HBx targeted miRNAs are classified as intragenic [25] and 15 of them display HBx peaks in the promoter region of their target genes. Independent anti-HBx ChIP experiments in HBV replicating HepG2 cells (Fig. 4a), HBV-infected NTCP-HepG2 cells (Additional file 1: Figure S5a) and PHHs (Additional file 1: Figure S5b) confirmed HBx binding to selected miRNA promoters (>4-fold enrichment relative to the control IgG ChIPed samples in 11 out of 13). Primers designed to overlap an HBx peak detected with a higher *P*-value (*P* = 1E-3) showed no enrichment in anti-HBx ChIPed DNA (Additional file 1: Figure S6).

The 75 miRNAs targeted by HBx include several miRNAs that have been implicated in the regulation of specific liver functions (*n* = 9), in HBV replication (*n* = 6), in hepatocarcinogenesis (*n* = 12), cancer (*n* = 30) and others the function of which is not yet known (*n* = 40) (Additional file 1: Table S2). The analysis of the selected miRNA promoter sequences bound by HBx using the Genomatix MatInspector resource showed the presence of binding sites for multiple transcription factors that have been reported to interact with HBx, including NFAT, CREB, NFkB, SREBP1, STAT, E2F and SMAD [4], a finding consistent with a mechanism of piggy-backing for HBx mediated by multiple transcription factors (Additional file 1: Table S3).

Next, we analyzed the impact of HBx chromatin recruitment on the expression of HBx targeted miRNAs. Figure 4b shows the differential expression, analyzed by real-time qPCR, of the 15 miRNAs encoded from the 13 promoters used for the independent validation of HBx chromatin recruitment in Fig. 4a. 8 miRNAs [miR-1913, miR-129-3p miR-129-5p, miR-26b; miR-551b-3p, miR-551b-5p, miR-3648, miR-3687] are consistently upregulated (>1.3 fold) and 5 miRNAs [miR-138, miR-224, miR-302e, miR-576-3p, miR-596] are downregulated (<0.7 fold) in HBV-wt HepG2 replicating cells. 4 miRNAs [miR-21, miR-26a-5p, miR-452, miR-552] are apparently not modulated or only slightly modulated by HBV replication in this assay (Fig. 4b). Notably, concordant results were obtained for 9/13 miRNAs included in the miRNAs Taqman microarrays profiles from HBV-infected (12 dpi) PHHs (Fig. 4c and Additional file 1: Figure S7). miR-21 and miR-452, that were only slightly up-regulated 48 h after transfection in HBV-replicating HepG2 cells, were significantly up-regulated in PHH 12 days after infection. HBV up-regulation of miR-21 expression is consistent with previous reports [26]. miR-552 was dowregulated in HBV-wt replicating HepG2 cells at 48 h withouth reaching <0.7 fold threshold but its levels were significantly reduced in HBV-infected PHH. miR-596 was dowregulated 48 h after transfection in HBV-wt replicating HepG2 cells and significantly upregulated in HBV-infected PHH.

HBx recruitment impacts on H4 histone acetylation of neighboring chromatin promoters. As shown in Fig. 4d, H4 acetylation was decreased on the promoter regions of miR-138-2, miR-224, miR-302e, miR-576-3p and miR-596, all repressed by HBx. Conversely, HBx recruitment was accompanied by an increased H4 acetylation at the miR-26b promoter (Fig. 4d), whose expression is activated in HBV-wt replicating HepG2 cells and HBV-infected PHH (Fig. 4b and Additional file 1: Figure S5). These results confirm the HBx ability to influence transcription by directly modulating the epigenetic status of target promoters and provide a mechanism for miRNA repression by HBx.

### A subset of HBx targeted miRNAs represses HBV replication

A number of miRNAs directly or indirectly promote or repress HBV replication (see ref. [27] and Additional file 1 : Table S4). Thus, miR-122 abrogates p53-mediated inhibition of HBV replication by targeting cyclin G1 and its interaction with p53 [28]. miR-372/373 and miR-501 promote HBV gene expression by targeting the transcription factor nuclear factor I/B [29] and HBXIP [30], respectively. miR-1 increases HBV transcription and replication by targeting HDAC4, which in turn represses HBV transcription [31]. miR-15b potentiates HBV replication by targeting HNF1a and relieving its repressive activity on HBV Enhancer 1 [32]. Conversely, miR-141 suppresses HBV expression and replication in HepG2 cells by targeting PPARa [33] and mir-130a inhibits hepatitis B virus replication by targeting PGC1α and PPARγ [34]. miR-199a-3p and miR-210 [35], miR-15a/miR-16-1 [36], the miR-17-92 cluster [37] and miR-1231 [38] have been shown to target HBV mRNAs directly and to inhibit HBV replication. None of these miRNAs showed HBx recruitment in our ChIP-Seq analysis, neither to their putative promoter sequence nor to the promoter regions of their host genes, with the exception of miR-15a and miR16.1 that are embedded into the DLeu2 lncRNA gene, the promoter of which harbors an HBx binding peak.

**Fig. 5:**
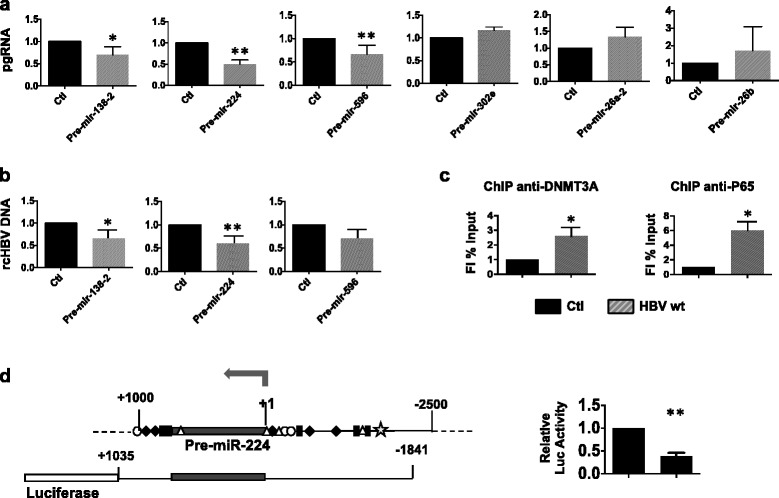
Impact of HBx-targeted miRNAs on 3.5Kb/pgRNA transcription and HBV replication. **a** HepG2 cells are co-transfected with linear wild type HBV genomes (HBV) and the indicated pre-hsa-miRNAs. HBV 3.5Kb/pgRNA levels were determined by real-time qPCR using specific primers. Results were expressed as fold induction relative to mock controls (Ctl) after normalization towards endogenous human β-actin mRNAs. **b** rcHBV-DNA levels associated to cytoplasmatic core particles were determined by real-time qPCR after normalization to β-globin. Histograms represent the mean from 3 independent experiments; *bars* indicate SDs. P values were determined using the Student’s *t* test. *P < 0.05. **c** DNMT3A and p65 recruitment on miR224 promoter. ChIPed chromatin from mock and HBV-wt replicating HepG2 cells. Results are expressed as in Fig. 4a. **d** *Left panel*. Putative regulatory region (−2500/+1000 from TSS) and schematic representation of the luciferase construct containing the miR-224 promoter region. The sequence was analyzed by motif software (http://www.genome.jp/tools/motif/) using a cut off score of 85%. *Black diamonds* represent p53 binding sites; white circles represent NFkB consensus; white triangles represent AP1 consensus; *black rectangles* represent TCF/LEF consensus and grey star represents HNF-3β consensus. *Right panel*. HepG2 cells were transfected with 250 ng of the −1841/+1035 miR-224 luciferase construct together with 250 ng of HBx expression vector. Luciferase activity was assayed 30h after transfection and expressed as fold induction over the control. Histograms represent the mean of 3 independent experiments each performed in duplicate; *bars* indicate S.D. P-values: * 0,02 ≤ *P* < 0,05; ** 0,01 ≤ *P* < 0.02; *** 0,005 ≤ *P* < 0.01

We investigated whether the miRNAs repressed by HBx binding to their regulatory regions might directly target HBV transcripts, and in particular the HBV pgRNA. HBx repression could relieve the miRNA-directed down-regulation of HBV replication and unveal new miRNA–dependent auto-regulatory loops in HBV replicating cells. *In silico* analysis revealed the presence of several putative seed sequences on the HBV genome specific for HBx-regulated miRNAs, which are also conserved across HBV genotypes (Additional file 1: Table S5). As shown in Fig. 5a, pre-miR-138, pre-miR-224 and pre-miR-596 overexpression reduces HBV pgRNA levels, whereas pre-miR-302e does not. Similarly, pre-mir-26a2, that is not modulated by HBV in our systems and we use as a control, did not affect HBV pgRNA levels (Fig. 5a). Notably, co-transfection of HBV-wt together with pre-miR-138-2, pre-miR-224, and pre-miR-596 resulted in a significant reduction of HBV replication, measured as cytoplasmic core particles associated rcHBV-DNA (Fig. 5b). Altogether, these results suggest that HBx repression of miR-138, miR-224 and miR-596 expression relieves the negative effects of these miRNAs on HBV replication. On the other hand, mir-302e likely downregulates HBV regulation indirectly, by targeting one or more genes involved in the regulation of HBV replication.

In order to better characterize the interplay between HBx, HBx-targeted miRNAs and HBV replication, we selected miR-224, whose expression is repressed by HBV replication (ref [39] bis and Fig. 4b and c) and increased in HCC patients [39–42] where its expression is driven by the TNF/LT-NFkB signaling [42]. Alignment of ChIP-Seq HBx occupancy on the intronic miR-224 regulatory region revealed the presence of a NFkB/p65 consensus motif, which is known to be a direct transcriptional regulator of miR-224 expression and up-regulation in human hepatocellular carcinoma [42]. HBx recruitment is accompanied by a strong co-recruitment of p65 and the DNMT3A DNA methyltransferase (Fig. 5c) in the same region of the miR-224 promoter, that is accompanied by an hypoacetylation of Histone 4 lysines (Fig. 4d). We also show that exogenously expressed HBx repressed miR-224 promoter in a transient luciferase reporter assay (Fig. 5d). Altogether these results strongly suggest the recruitment of HBx as part of a transcriptionally inactive NFkB/p65 complex on the miR-224 promoter, which is responsible for the silencing of miR-224 transcription.

**Fig. 6:**
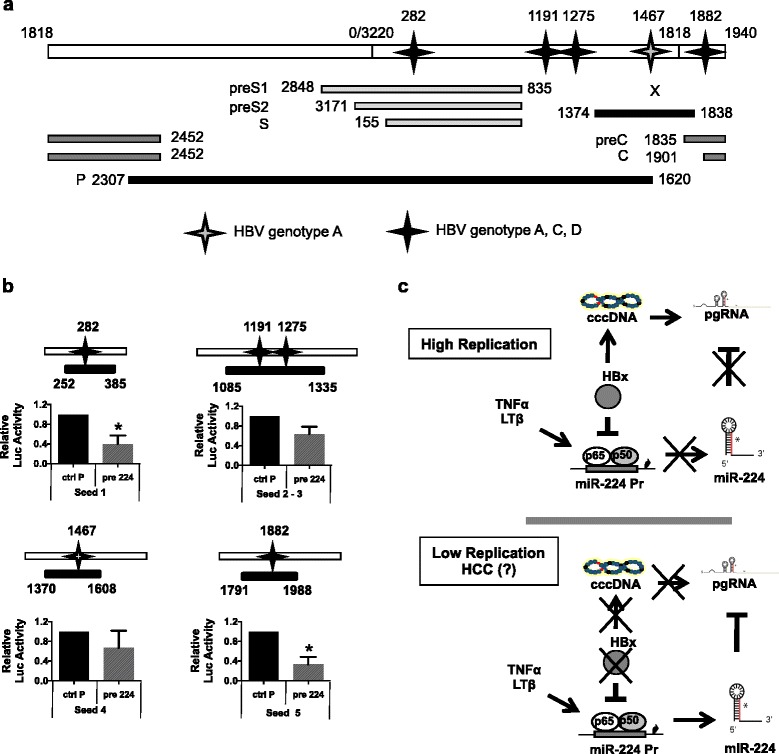
Direct targeting of HBV 3.5Kb/pgRNA by miR-224. **a** In silico analysis of the HBV genome, performed with the RNAhybrid tool on the Bielefeld University Bioinformatics Server, revealed the presence of several putative miR-224 seed sequences that are conserved across HBV genotypes. **b** *Upper panels*. Schematic representation of the putative seed sequences of HBV genome cloned on the 3′ UTR of the Renilla luciferase gene in the pRL-TK vector (Promega). *Lower panels*. HepG2 cells were transfected with the above indicated luciferase constructs together with 30nM of pre-miR-224 or its relative control. Luciferase activity was assayed 30h after transfection and expressed as fold induction over the control. All histograms represent the mean of three indipendent experiments each performed in duplicate; *bars* indicate S.D. P-values: * 0,02 ≤ *P* < 0,05; ** 0,01 ≤ *P* < 0.02; *** 0,005 ≤ *P* < 0.01. Grey stars, HBV genotype A; *black stars*, genotype A, C, D. **c** Proposed model for a differential modulation of miR-224 expression by HBx and inflammatory cytokines in ealrly phases of HBV infection and in HCC

Next, to confirm that the inhibition of HBV replication by miR-224 is indeed the result of a direct targeting of HBV 3.5Kb/pgRNA, the 5 putative seed sequences identified by the RNAhybrid software in the HBV genome (Fig. 6a) were cloned at the 3′UTR of the Renilla Luciferase gene in the pRL-TK vector (Fig. 6b). Co-transfection of the different Renilla Luciferase constructs together with pre-miR-224 showed a significative inhibition of the Luciferase activity for constructs containing seed 1 and seed 4, thus confirming that HBV is a direct target of miR-224 which inhibits viral replication by directly binding HBV genome on seed 1 and seed 4 (Fig. 6b).

## Discussion

Our ChIP-Seq genome wide analysis of HBx chromatin recruitment in HBV replicating cells provides a repertoire of genes and ncRNAs directly targeted by HBx and led us to propose new mechanisms by which HBx potentiates HBV replication. As already mentioned, a possible limitation of our study is that our HBx ChIP-Seq data set was generated in HBV replicating HepG2 cells, which are transformed cells derived from primary liver tumor, wild type for p53 and mutated for β-catenin. Indeed, some of the “enriched genes” are associated with pathways, such as metabolism, chromatin dynamics and cancer, that are often found to be deregulated in transcriptomic analysis of immortalized and tumor cell lines. It cannot be excluded that a number of HBx genomic binding sites identified in our study might reflect a bias for transcriptionally active chromatin regions present in HepG2 cells. It is conceivable that the same regions might not be transcriptionally active, and therefore potentially not be targeted by HBx, in normal hepatocytes (i.e. a human liver recently infected by HBV or primary human hepatocytes infected in vitro by HBV). However, strong clinical evidence links HBV replication with the development of HBV-related HCCs and HBx expression has been widely implicated in HCC development and progression. Thus, a repertoire of HBx genomic binding sites identified in a tumor derived hepatocytic cell line, such as the HepG2 cells, might be very relevant for HBV-induced hepatocarcinogenesis. Most HBx targets validated in our study have been confirmed in primary hepatocytes from different donors and, notably, HBx is recruited very early post-infection on the regulatory sequence of miR-21, a well established *onco-miR*, also in HBV-infected primary human hepatocytes.

**Fig. 7:**
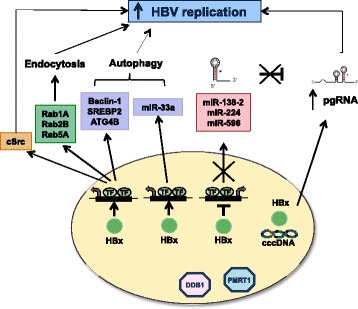
Schematic representation of HBx-dependent mechanisms that contribute to boost HBV replication

We found that HBx activates several genes and miRNAs that potentiate endocytosis and autophagy to favor HBV replication, and represses miRNAs (miR-224, miR-138 and miR-596) that potentially target the HBV pgRNA and would inhibit HBV replication (Fig. 7). Thus, HBx binding to the cccDNA increases pgRNA transcription and HBV replication and, at the same time, HBx protects the pgRNA from the negative effects of miRNAs miR-224, miR-138 and miR-596 by targeting their promoters and inhibiting their expression. To better characterize the interplay between HBx, HBx targeted miRNAs and HBV replication we focused on miR-224. The down-regulation of miR-224 expression in HBV replicating cells (ref [35] and Fig. 4b and c) and HBV infected primary hepatocytes (Additional file 1: Figure S5) is in apparent contradiction with the reported up-regulation of miR-224 in elevated in HCC [40–42] including HBV-related HCCs [42]. Notably, Amaddeo et al. have reported that HBx inactivating mutations, including missense/stop and frameshift mutations as well as deletions, are selected in HBV-related HCCs that carry a lower number of viral copies as compared to the non-tumor tissues [43]. Although it would be challenging to obtain a formal demonstration in vivo, it is tempting to speculate that, in the early phases of HBV infection, HBx binding at or near the p65/NFkB sites in the miR-224 promoter leads to the repression of miR-224 expression, that would be detrimental for viral replication, whereas HBx inactivation leaves the miR-224 promoter free to respond to TNF/LT-NFkB signaling in HCCs and highly displastic nodules.

## Conclusions

The analysis of HBx genome wide chromatin recruitment provides a repertoire of genes and ncRNAs directly targeted by HBx and led to the identification of new mechanisms by which HBx positively regulates cccDNA transcription and HBV replication.

## Methods

### Cell cultures

HepG2 (ATCC HB-8065), HepG2.2.15 (a HepG2 clone transfected with a plasmid containing two head-to-tail dimers of the HBV genome, kindly provided by H. Will, MPI, Martinsried, Germany) and NTCP-HepG2 (a HepG2 clone transfected with a plasmid expressing the HBV receptor Na^+^-taurocholate cotransporting polypeptide (NTCP), kindly provided by S Urban, Heidelberg University, Germany) cells were cultured in supplemented Dulbecco’s modified Eagle’s medium (DMEM) as described [44] and maintained in a 5% CO2 humidified incubator at 37 C.

### Transient transfection of full-length HBV DNA genomes

The pCR.HBV.A.EcoRI and the pCR.HBXmt.A.EcoRI plasmids were restricted by EcoRI-PvuI (New England Biolabs) to generate monomeric linear full-length WT (genotype A, adw) and HBx-mt (carrying a termination site at the codon 8 of the X ORF) HBV genomes [44]. Linear HBV monomers were transfected into HepG2 human hepatoma cells using the Mirrus Bio transIT-LT1 (Mir 2300A). Briefly, 2–3 × 10^6^ or 1–2 × 10^7^ HepG2 cells are seeded in 100-mm or 150-mm-diameter Petri dishes, respectively, transfected after 24 h with 1 to 2 μg of digested HBV DNA and harvested 48 h post-transfection. A GFP expression vector (500 ng) was included to assess transfection efficiency (range 28%–32%). The HBV region spanning the predicted ends of the linear dsDNA was amplified and sequenced to exclude the generation of circular HBV DNA molecules carrying sequence modifications at the recombination site. Southern blot analysis has shown that the HBV cccDNA species in the nuclear extracts from HepG2 transfected cells co-migrate with the cccDNA isolated from an HBV-infected liver used as a positive control and are converted into a linear DNA by XhoI digestion [18].

### Primary culture of human hepatocytes and HBV infection

Primary human hepatocytes (PHH) (provided by Prof. M. Rivoire) were prepared from HBV, HCV and HIV negative adult patients undergoing lobectomy or segmental liver resection for medically required purposes unrelated to this research program. PHH were prepared and cultured as described elsewhere [45]. Briefly, the cells are plated overnight in collagen-coated dishes (BD Biosciences) at 2×10^5^ cells/cm^2^ in William’s medium (Life Technologies) supplemented with 10% FetalClone II (Thermo Scientific), 1% penicillin/streptomicine and 1% glutamine (Life Technologies), as well as 5 μg/ml Insulin and 5×10^-7^ M hydrocortisone hemisuccinate (Sigma Aldrich). After 24 h PHH are extensively washed in serum-free medium and kept in serum-free medium for one more day to counter select the growth of contaminating fibroblast and endothelial cells, and infected 48 hours after plating with HBV virus (i.e. inoculum) produced in HepG2.2.15 cells [46].

### ChIP assays

Cells are resuspended in 0,2–0,4 ml of ChIP lysis buffer (50 mM Tris HCL, pH 8, 0.5% NP40, 1 mM EDTA, and 100 mM NaCL), incubated 10 min at 4°C and the lysate centrifuged at 10,000 g for 2 min. The nuclei are fixed in 1% formaldehyde for 30 min at 4°C, extracted with a 20 mM Tris, pH 8, 3 mM MgCl2, 20 mM KCl buffer containing protease inhibitors, pelleted by microcentrifugation and lysed in SDS lysis buffer (1% sodium dodecyl sulfate, 10 mM EDTA, 50 mM Tris-chloride, pH 8.1) containing protease inhibitors. Chromatin is sonicated using a Bioruptor Sonicator (Diagenode Inc) to generate 300- to 1000-bp DNA fragments (5 pulses of 45 s at 80% power) for ChIP and 200- to 300-bp DNA fragments (total time of 20 min, 30 s ON, 30 s OFF) for ChIP-Seq. After microcentrifugation, the supernatant is diluted 1:10 in dilution buffer (0.01% sodium dodecyl sulfate, 1.1% Triton X-100, 1.2 mM EDTA, 16.7 mM Tris-chloride, pH 8.1, 167 mM NaCl, containing protease inhibitors). 100 μl of antibody-coupled magnetic beads (Invitrogen Dynalbeads) are added to each 1 ml chromatin preparation and incubated on a rotator for 14–16 h at 4°C. The antibodies used are anti-HBx (MA1-081; mouse monoclonal IgG1), anti-AcH4 (06–866, Upstate; rabbit polyclonal IgG recognizing histone H4 acetylated at lysines 6, 9, 13, and 17), anti-DNMT3A (sc-20703; Santa Cruz Biotechnology Inc.), anti-p65 (sc-372; Santa Cruz Biotechnology Inc.). Nonspecific immunoglobulins (Santa Cruz Biotechnology Inc.) are used as negative controls. After the reverse cross-linking, immunoprecipitated chromatin was purified by phenol/chloroform (1:1) extraction and ethanol precipitation. ChIPed chromatin is analyzed by real-time PCR amplification using either primers (NCC1 and CCCAS) and probes (FL and Red) specific for the HBV cccDNA [3] or specific primers for genes and miRNAs promoters and the SYBR Green DNA Master mix (Applied Biosystems, Inc., Foster City, US-CA) (Additional file 1: Table S6). For ChIP-Seq chromatin is prepared from ~2×10^7^ cells / experimental point and processed according to the Illumina ChIp-Seq libraries generation protocol (IP-102-1001) and sequenced on Illumina GAII/GAIIx platforms. The eluate was quantified by using a Qubit (Invitrogen) fluorometer. The quality of each anti-HBx ChIP processed for ChIP-Seq was prealably assessed by controlling HBx binding to the HBV cccDNA [8] and to the MT1F gene promoter [47]. DNA fragments recovered from reverse cross-linked chromatin are repaired, ligated to adapters, size selected and PCR-amplified to generate NGS libraries following the Illumina DNA Library Construction Kit.

### ChIP-Seq data analysis

Reads (36 bp) were mapped to the UCSC human genome hg19 using Bowtie (version 0.12.9) [48]. Uniquely aligned reads with no mismatches were retained for subsequent analyses. Peak-calling was performed using model-based analysis for ChIP-seq (MACS, version 1.4.2) [49], following the protocol described in Feng et al. [50]. Peak-calling was performed against a reference input sample from the same HepG2 cell line. Home-made Perl scripts (www.biocomputing.it; “Tools” section; Scripts related to Guerrieri et al.) were used to locate HBx candidate binding sites along the genome, taking into account three categories: promoters (10 kb upstream or 1 kb downstream the transcriptional start site (TSS) of genes), intragenic (from 1 kb after TSS up to the transcript end site (TES)) and intergenic. For microRNAs, in particular, the promoter HBx binding sites were located within the range of (−5000; +1000) bps relative to the TSS. All potential target protein-coding genes were annotated according to UCSC, RefSeq and GenCode; for non-coding genes (snoRNAs, snRNAs and lincRNAs) we referred to the Ensembl Annotation and for microRNAs to the miRBase database v18 [51].

### Functional enrichment analyses of candidate HBx target genes

All protein-coding genes with putative HBx binding sites (promoters or intragenic) were analysed by DAVID [52] and FIDEA [53] web servers using KEGG [54] and Reactome pathways [55, 56], the InterPro families [57] and the Gene Ontology (GO) Biological Processes [58]. Only biological terms with Benjamini-corrected *p*-value < 0.05 were considered enriched.

### Plasmids and luciferase assay

The miR-224 promoter region −1841/+1035 (relative to the pre-miR sequence was amplified by PCR from HepG2 genomic DNA with the following primers: luc-miR-224_FOR 5′-AATCCTGTGCACCTCATCCTCTGT-3′; luc-miR-224_REV 5′-GACGAGCGGAG AAGGTTCTT-3′. The amplified region was cloned into the pGL3 Basic Vector (Promega, Madison, WI, USA) using the T4 DNA ligase (Promega, Madison, WI, USA) and the construct was confirmed by sequencing. Putative miR-224 seed sequences in the HBV genome (genotipe A; ADW) were amplified by PCR with specific primers (Additional file 1: Table S6), cloned in the 3′ UTR of the Renilla luciferase gene in the pRL-TK vector (Promega) and confirmed by sequencing. For luciferase assay, cells are transfected with the indicated luciferase reporter constructs and expression vectors using the Lipofectamine Plus reagent (Invitrogen Inc., Carlsbad, CA, USA). Luciferase activity in cell lysates is measured using the Luciferase Assay System (Promega, Madison, WI, USA) 30 h after transfection and the results normalized for protein levels (Bradford, Bio-Rad Laboratories, Hercules, CA, USA). Pre-miRNA-224 and anti-miRNA-224 (Ambion #AM17100 and #AM17000) are transfected with the Lipofectamine plus reagent (Invitrogen Inc., Carlsbad, CA, USA) at a final concentration of 30 nmol/l and cells were analyzed 30 h after transfection.

### HBV pgRNA and cellular mRNA analysis

Total RNA was extracted using the TRIzol reagent (Invitrogen). RNA samples are treated with RQ1 RNase-Free DNase (Promega) for 30 min at 37°C and RNA quality and quantity monitored by ethidium bromide staining and UV absorbance. For pgRNA analysis, 2 μg of DNase-treated RNA are reverse transcribed (ThermoScript RT-PCR System, Invitrogen) and quantified by real-time PCR analysis (Light Cycler; Roche Diagnostics) using the following pgRNA-specific primers and probes: forward primer, 5′-GCCTTAGAGTCTCCTGAGCA-3′, reverse primer, 5′-GAGGGAGTTCTTCTTCTAGG-3′, FRET hybridization probes, 5′–AGTGTGGATTCGCACTCCTCCAGC-FL-3′, and Red640-5′ATAGACCACCAAATGCCCCTATCTTATCAAC-3′. RNA samples are normalized using the hGAPDH housekeeping gene Light Cycler Set (Roche Diagnostics). miRNAs and mRNAs levels in cultured cells were assayed, after reverse transcription (ThermoScript, Invitrogen, Inc., Carlsbad, US-CA) using miRNA specific stem-loop primers or random primers, by quantitative qPCR using either a specific TaqMan FAM-probe (Applied Biosystems, Inc.) with the specific primers. Results were normalized with respetct to RNU38 or β-actin and expressed as described above.

### Taqman® array microRNA cards

Total RNA is extracted from PHH at different time points after HBv infection with TRIzol® reagent (Life Technologies) according to manufacturer’s instructions. The Megaplex™ RT primer human pool A + B v3.0 (Life Technologies) was used for miRNA-specific reverse transcription. RNA quality and quantity were monitored by ethidium-bromide staining and by UV absorbance. The Megaplex™ RT product was then loaded on Taqman® microRNA cards A + B and run performed with default thermal cycling conditions (Taqman® array user bulletin – Life Technologies). Data were collected through SDS 2.3 software and analyzed through RQ Manager 1.2 program (Life Technologies).

### Core-particles associated HBV DNA purification and quantitation

Cells are washed with ice-cold PBS, lysed in 50 mmol Tris–HCl, pH 7.4, 1 mmol EDTA, and 1% NP-40 (lysis buffer A) and nuclei pelleted by centrifugation for 1 min at 10,000 g. The supernatants are adjusted to 100 mmol MgCl2, treated with 100 mg/ml of DNase I for 30 min at 37°C and then with 0.5 mg/ml proteinase K and 1% SDS for 2 h at 50°C. Nucleic acids are purified by phenol- chloroform (1:1) extraction and ethanol precipitation adding glycogen. HBV DNA is quantified by real-time PCR in a Light Cycler instrument (Roche) using the following primers and probes: forward, 5′-CTCGTGGTGGACTTCTCTC-3′, and reverse 5′-CAGCAGGATGAAGAGGAA-3′. We also used specific FRET hybridization probes: 5′-CACTCACCAACCTCCTGTCCTCCAA-FL-3′, Red640, 5′-TGTCCTGGTTATCGCTGGATGT GTCT-3′. Amplifications were performed as follows: 95°C for 5 min, followed by 45 cycles at 95°C for 10 s, 58°C for 10 s, and 72°C for 20 s.

### Immunofluoresence and transferrin-uptake assay

HepG2 cell lines were grown overnight on glass coverslips in 6-well plates. The cells were serum-starved at 37 °C for 1 h in starving medium (DMEM 0%), then incubated at 37 °C for 15–20 min in starving medium containing 25 μg/ml of biotinylated holo-transferrin (Sigma T-3915). Cells were fixed in 4% paraformaldehyde at room temperature for 20 min, washed thrice with PBS, permeabilised, blocked with TBS + 0.2% Saponin + 10% serum anti rabbit for 15 min, and then incubated at 37 °C for 1 h with 1:500 dilution of Streptavidin from Molecular Probes conjugated to the fluorophor Alexa 594 (Invitrogen). After three washes with TBS + 0.02% Saponin + 1% serum, the coverslips were mounted onto glass slides. Images of Transferrin, DAPI and GFP-fluorescence were acquired using a *Zeiss 510 Meta* confocal microscope (all images were obtained with the same exposure setting). Alexa 594-conjugated transferrin signal intensity was quantified for 10 transfected cells in each set, and the mean intensity calculated and normalized against that of the nontransfected cells (Mock) with the software ImageJ.

### Statistics

P values were determined using the 2-tailed Student’s *t* test. Wilcoxon rank-sum test was used for nonparametric pair-wise comparisons. *P* < 0.05 was considered significant.

## Additional file

Additional file 1: Figure S1. HBx ChIP-Seq experiments analysis. **Figure S2.** HBx occupancy on cellular promoters. **Figure S3.** HBx occupancy on the control RFP2 promoter. **Figure S4.** HBx occupancy on cellular promoters in NTCP-HepG2 cells infected with wild-type and mt-HBx HBV. **Figure S5.** HBx occupancy on miRNAs promoters in NTCP-HepG2 cells and PHHs infected with HBV-wt virus. **Figure S6.** ChIP validation of high P value HBx peak. **Figure S7.** HBx targeted miRNAs expression in HBV-infected Primary Human Hepatocytes. **Table S1.** Rab family and ATG family. **Table S2.** HBx targeted miRNAs functions (PubMed). **Table S3.** TF binding sites overlapping HBx peaks (Genomatix MatInspector). **Table S4.** miRNAs that activate or repress HBV replication HBV replication. **Table S5.** miRNA seeds predicted in HBV pgRNA sequence and conserved across HBV genotypes. **Table S6.** List of primers used thoughout the manuscript. (PDF 647 kb)

## Abbreviations

ATG: Autophagy related genes
ChIP-Seq: Chromatin immunoprecipitation sequencing
DDB1: DNA damage-binding protein 1
DNMT3A: DNA Methyltransferase 3 Alpha
HBV: Hepatitis B Virus
HCC: Hepatocellular carcinoma
lncRNAs: long non-coding RNAs
miRNAs: microRNAs
ncRNAs: non-coding RNAs
NTCP: Na + −taurocholate cotransporting polypeptide
pgRNA: pregenomic RNA
PHH: Human primary hepatocytes
PRMT1: Protein arginine N-methyltransferase
RAB: Ras-related in brain GTPase family of genes
TSS: Transcription start site
