# Supplementary material for: Genome-wide identification of direct HBx genomic targets
Source: BMC Genomics. 2017 Feb 17;18:184. doi: 10.1186/s12864-017-3561-5 (PMC5316204; doi:10.1186/s12864-017-3561-5)
Supplement: Additional file 1: Figure S1. — HBx ChIP-Seq experiments analysis. Figure S2. HBx occupancy on cellular promoters. Figure S3. HBx occupancy on the control RFP2 promoter. Figure S4. HBx occupancy on cellular promoters in NTCP-HepG2 cells infected with wild-type and mt-HBx HBV. Figure S5. HBx occupancy on miRNAs promoters in NTCP-HepG2 cells and PHHs infected with HBV-wt virus. Figure S6. ChIP validation of high P value HBx peak. Figure S7. HBx targeted miRNAs expression in HBV-infected Primary Human Hepatocytes. Table S1. Rab family and ATG family. Table S2. HBx targeted miRNAs functions (PubMed). Table S3. TF binding sites overlapping HBx peaks (Genomatix MatInspector). Table S4. miRNAs that activate or repress HBV replication HBV replication. Table S5. miRNA seeds predicted in HBV pgRNA sequence and conserved across HBV genotypes. Table S6. List of primers used thoughout the manuscript. (PDF 647 kb) [file 12864_2017_3561_MOESM1_ESM.pdf]

Supplementary Files

Genome-wide identification of direct HBx targets that control HBV replication

Francesca Guerrieri [1, 2], Laura Belloni [1, 2], Daniel D'Andrea [3], Natalia Pediconi [4], Loredana Le Pera[1, 3], Barbara Testoni [4], Cecilia Scisciani [2], Oceane Floriot [5], Fabien Zoulim [5], Anna Tramontano [1,3, 6], Massimo Levrero [1, 2, 5]

[1] *Center for Life NanoScience@Sapienza, Istituto Italiano di Tecnologia, Rome, Italy*
[2] *Dept of Internal Medicine - DMISM, Sapienza University, Rome Italy*
[3] *Biocomputing Lab, Department of Physics, Sapienza University of Rome, Italy*
[4] *Dept of Molecular Medicine, Sapienza University, Rome, Italy*
[5] *INSERM U1052, Cancer Research Center of Lyon (CRCL), Lyon, 69008, France*
[6] *Istituto Pasteur Fondazione Cenci Bolognetti, Rome, Italy*

Tabel of contents:

pages 2-7: Supplementary Figures

pages 8-14: Supplementary Tables

a

Exp	Name	#Good unique reads	#Uniquely mapped reads	%Uniquely mapped reads
SAP	Input	33,167,085	25,687,082	77
SAP	HBx_wt_1	29,109,646	21,261,284	73
SAP	HBx_wt_2	13,090,991	7,994,287	61
IFO	Input	17,516,410	12,157,995	69
IFO	HBx_wt_1	17,148,341	7,996,242	47
IFO	HBx_wt_3	15,629,897	7,141,652	46

b

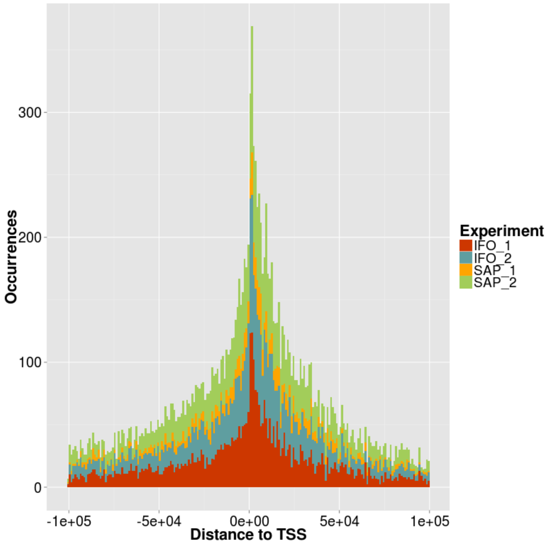

c

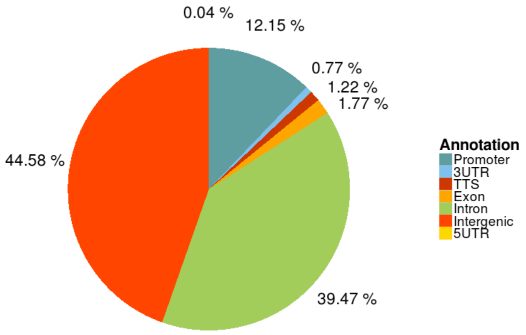

d

Hsa-miR	fdr	Hsa-miR	fdr	Hsa-miR	fdr	Hsa-miR	fdr
**Hsa-miR-224**	**0**	Hsa-miR-3139	0.24	Hsa-miR-4698	0.76	Hsa-miR-3617	1.75
**Hsa-miR-452**	**0**	Hsa-miR-548p	0.24	Hsa-miR-640	0.79	Hsa-miR-944	1.80
**Hsa-miR-21**	**0**	Hsa-miR-302e	0.25	Hsa-miR-4740	0.80	**Hsa-miR-26a-2**	**2.14**
**Hsa-miR-3648**	**0**	Hsa-miR-663a	0.29	Hsa-miR-4662b	0.80	Hsa-miR-4488	2.15
**Hsa-miR-576**	**0.14**	Hsa-miR-138-2	0.30	Hsa-miR-5698	0.82	Hsa-miR-3197	2.26
**Hsa-miR-1913**	**0.14**	**Hsa-miR-551b**	**0.36**	Hsa-miR-4681	0.82	Hsa-miR-4770	2.42
**Hsa-miR-26b**	**0.15**	Hsa-miR-5095	0.47	Hsa-miR-584	0-84	Hsa-miR-543	2.76
Hsa-miR-5588	0.15	Hsa-miR-4682	0.51	Hsa-miR-4648	0.87	Hsa-miR-495	2.76
Hsa-miR-3691	0.15	Hsa-miR-1539	0.55	Hsa-miR-3650	0.88	Hsa-miR-4664	3.54
Hsa-miR-4703	0.16	Hsa-miR-4429	0.59	Hsa-miR-133a-2	0.89	Hsa-miR-4317	3.73
Hsa-miR-5193	0.16	Hsa-miR-663b	0.59	Hsa-miR-3914-1	0.95	Hsa-miR-4321	3.87
Hsa-miR-943	0.16	Hsa-miR-663a	0.63	Hsa-miR-3170	0.95	**Hsa-miR-3648**	**11.1**
**Hsa-miR-129-1**	**0.16**	Hsa-miR-4666a	0.67	Hsa-miR-548a-2	1.04	**Hsa-miR-3687**	**11.1**
**Hsa-miR-552**	**0.17**	**Hsa-miR-3648**	**0.69**	Hsa-miR-3622b	1.18	Hsa-miR-4448	37.5
Hsa-miR-3657	0.17	**Hsa-miR-3687**	**0.69**	Hsa-miR-4286	1.22	Hsa-miR-3916	37.6
Hsa-miR-1973	0.17	Hsa-miR-639	0.69	Hsa-miR-555	1.23	Hsa-miR-4442	37.9
Hsa-miR-4276	0.17	**Hsa-miR-596**	**0.70**	Hsa-miR-4429	1.24	Hsa-miR-4446	38.0
Hsa-miR-3909	0.18	Hsa-miR-4501	0.71	Hsa-miR-4425	1.25	Hsa-miR-4438	43.1
Hsa-miR-4476	0.18	Hsa-miR-378g	0.73	Hsa-miR-4309	1.29		
Hsa-miR-1244-2	0.20	Hsa-miR-3667	0.73	Hsa-miR-639	1.73		
Hsa-miR-626	0.22	Hsa-miR-4710	0.74	Hsa-miR-5006	1.74		

Figure S1. HBx ChIP-Seq experiments analysis. Libraries were sequenced using the Illumina Genome Analyzer II (two IFO libraries) or the Genome Analyzer IIx (two SAP libraries). Total input DNA from each experiment served as control sample. *a)* Number of reads obtained in the different experiments; *b)* Histogram showing the distribution of peaks with respect to the Transcription Start Site (TSS); *c)* Distribution of HBx peaks in the different genomic regions. *d)* list of the 75 HBx targeted miRNA promoters sorted according to their False Discovery Rate (FDR).

a **b**

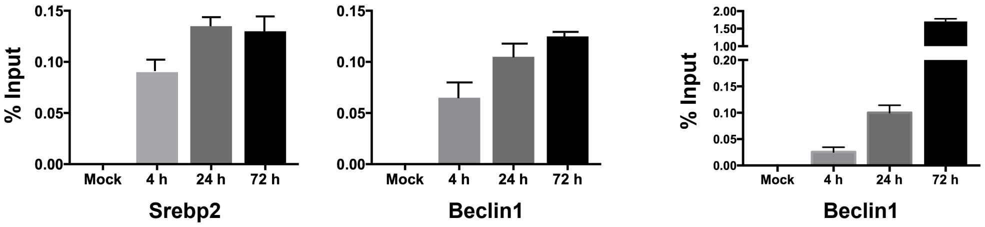

Figure S2. HBx occupancy on cellular promoters in: a) NTCP-HepG2 cells and b) PHHs infected with HBV-wt virus. Anti-HBx ChIPed chromatin from NTCP-HepG2 cells (a) and PHHs (b) infected with HBV-wt virus is analyzed with primers specific for the SREBP2 and Beclin-1 promoters. ChIP results are expressed as % of Input. Histograms show the mean from two independent experiments; bars indicate SD.

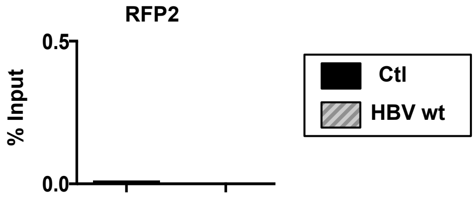

Figure S3. HBx occupancy on the control RFP2 promoter. HBx occupancy on the RFP2 promoter showed no HBx enrichment in independent ChIP experiments. Results are expressed as in Figure S2.

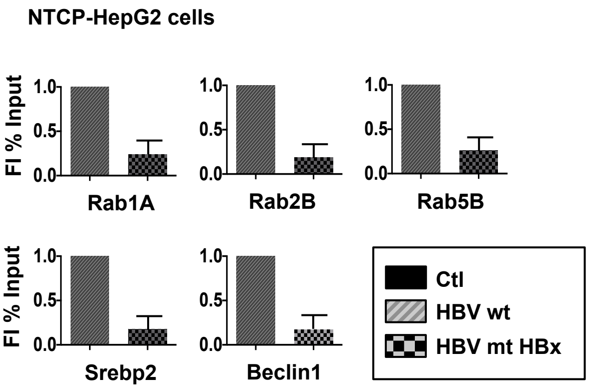

Figure S4. HBx occupancy on cellular promoters in NTCP-HepG2 cells infected with wild-type and mt-HBx HBV. Anti-HBx ChIPed chromatin from NTCP-HepG2 cells infected with HBV-wt and HBV-mt-HBx virus is analyzed with primers specific for the RAB1A, RAB2B, RAB5B, Beclin-1 and SREBP2 promoters. ChIP results are expressed as Fold Induction (FI) of the % of Input. Histograms show the mean from three independent experiments; bars indicate SD.

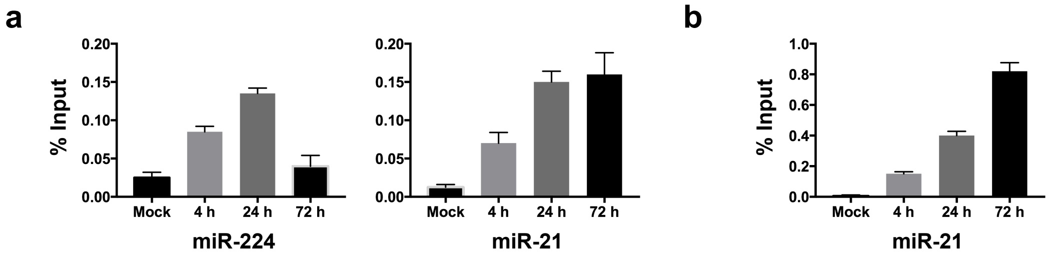

Figure S5. HBx occupancy on miRNAs promoters in: a) NTCP-HepG2 cells and b) PHHs infected with HBV-wt virus. Anti-HBx ChIPed chromatin from NTCP-HepG2 cells (a) and PHHs (b) infected with HBV-wt virus is analyzed with primers specific for the miR-224 and miR-21 promoters. Results are expressed as in Figure S2.

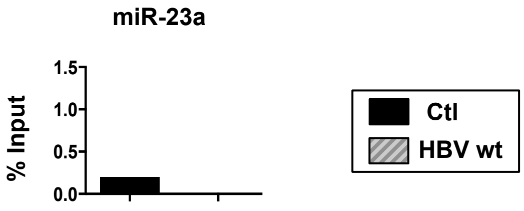

Figure S6. ChIP validation of high P value HBx peak. HBx occupancy on miR-23a (peak P value =1E-3) showed no enrichment in anti-HBx in an independent ChIP validation experiment. Results are expressed as in Figure S2.

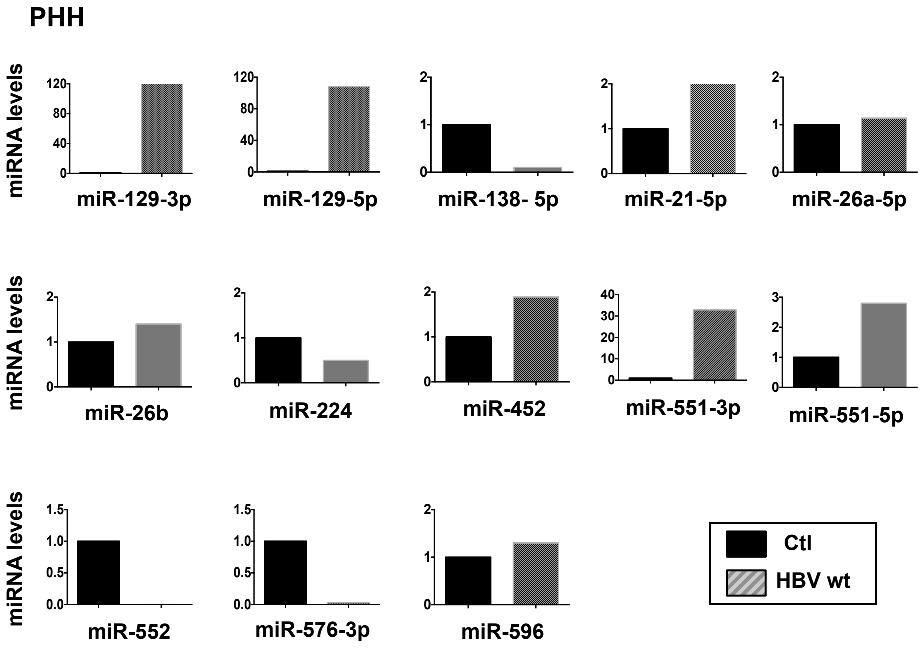

Figure S7. HBx targeted miRNAs expression in HBV-infected Primary Human Hepatocytes. miRNAs expression was analysed by Taqman PCR-arrays in HBV infected (12 dpi) Primary Human hepatocytes (PHH).

Table S1

Rab family & ATG family table

Genes	Location
RAB1A, RAB2B, RAB5A, RAB6C, RAB8A, RAB12, RAB27A, RAB28, RAB31, RAB38, RAB11FIP2, RAB11FIP3	Promoter
RAB3B, RAB3C, RAB3D, RAB4A, RAB5B, RAB8BA, RAB8B, RAB10, RAB18, RAB27A, RAB28, RAB31P, RAB37, RAB32, RAB11FIP3, RAB11FIP5	In gene
ATG4B	Promoter
ATG3, ATG4B, ATG5, ATG7, ATG9A, ATG10, ATG16L1, ATG9B	In gene

Table S2

HBx targeted miRNAs functions (PubMed)

Gene	Liver	HCC	HBV	Cancer	NA	Other
hsa-mir-1244-2						X
hsa-mir-129-1	X	X	X	X		
hsa-mir-133a-2		X		X		
hsa-mir-138-2		X	X	X		
hsa-mir-1539				X		
hsa-mir-1913					X	
hsa-mir-1973				X		
hsa-mir-21	X	X	X	X		
hsa-mir-224	X	X	X	X		
hsa-mir-26a-2	X	X	X	X		
hsa-mir-26b	X	X		X		
hsa-mir-302e					X	
hsa-mir-3139					X	
hsa-mir-3170				X		
hsa-mir-3197						X
hsa-mir-3617						X
hsa-mir-3622b					X	
hsa-mir-3648						
hsa-mir-3648						
hsa-mir-3648				X		
hsa-mir-3650					X	
hsa-mir-3657					X	
hsa-mir-3667					X	
hsa-mir-3687					X	
hsa-mir-3691					X	
hsa-mir-378g				X		
hsa-mir-3909					X	
hsa-mir-3914-1					X	
hsa-mir-3916					X	
hsa-mir-4276					X	
hsa-mir-4286				X		
hsa-mir-4309					X	
hsa-mir-4317				X		
hsa-mir-4321					X	
hsa-mir-4425					X	
hsa-mir-4429						X
hsa-mir-4438					X	
hsa-mir-4442					X	
hsa-mir-4446				X		

Gene	Liver	HCC	HBV	Cancer	NA	Other
hsa-mir-4448	X			X		
hsa-mir-4476					X	
hsa-mir-4488					X	
hsa-mir-4501				X		
hsa-mir-452	X	X		X		
hsa-mir-4648					X	
hsa-mir-4662b				X		
hsa-mir-4664					X	
hsa-mir-4666a					X	
hsa-mir-4681					X	
hsa-mir-4682					X	
hsa-mir-4698					X	
hsa-mir-4703					X	
hsa-mir-4710					X	
hsa-mir-4740					X	
hsa-mir-4770					X	
hsa-mir-495		X		X		
hsa-mir-5006					X	
hsa-mir-5095					X	
hsa-mir-5193					X	
hsa-mir-543	X			X		
hsa-mir-548a-2						X
hsa-mir-548p					X	
hsa-mir-551b		X	X			
hsa-mir-552				X		
hsa-mir-555					X	
hsa-mir-5588					X	
hsa-mir-5698					X	
hsa-mir-576				X		
hsa-mir-584	X			X		
hsa-mir-596		X		X		
hsa-mir-626						X
hsa-mir-639				X		
hsa-mir-640				X		
hsa-mir-663a				X		
hsa-mir-663b				X		
hsa-mir-943		X				
hsa-mir-944				X		

Table S3

TF binding sites overlapping HBx peaks
(Genomatix MatInspector)

	NFAT	CREB	NFkB	p53	STAT	E2F	SMAD
**miR-224**	■	■	■	■	■	■	
**miR-129-1**			■	■		■	
**miR-138-2**	■	■				■	■
**miR-302e**		■		■		■	
**miR-596**	■	■	■		■	■	■
**miR-26a-2**		■	■		■		■
**miR-26b**			■			■	■
**miR-551b**		■			■	■	
**miR-552**		■	■	■			■
**miR-1913**	■						
**miR-576**	■	■					
**miR-3648**		■	■		■	■	
**miR-21**	■	■	■	■	■	■	■

Table S4

miRNAs that activate or repress HBV replication

	HBV replication	Target / mechanisms	References
miR-1	up	HDAC4	23
miR-15a<br>miR-16-1	down	Viral RNAs	28
miR-15b	up	HNF1a	24
miR-17-92 cluster	down	Viral RNAs	29
miR-122	down	Cyclin G1-p53	20
miR-130a	down	PGC1a-PPARg	26
miR-141	down	PPARa	25
miR-372/373	up	nuclear factor I/B	21
miR-199a-3p<br>miR-210	down	Viral RNAs	27
miR-1231	down	Viral RNAs	30
miR-501	up	HBXIP	22

Table S5

miRNA seeds predicted in HBV pgRNA sequence and conserved across HBV genotypes (RNA-hybrid a)

miRNA	Seeed 5p	Seed 3p
21	-22,2 kcal/mol	-21,4 kcal/mol
26a-2	-17,8 kcal/mol	-19,6 kcal/mol
26b	-21,5 kcal/mol	-25,4 kcal/mol
129-1	-24,5 kcal/mol	-27,9 kcal/mol
138-2	-31,2 kcal/mol	-22,3 kmol/cal
224	-27 kcal/mol	-25,8 kcal/mol
302e	-22,6 kcal/mol	/
452	-25,6 kcal/mol	-19,7 kcal/mol
551b	-26,2 kcal/mol	-24,8 kmol/cal
552	-25 kcal/mol	/
576	-22,2 kcal/mol	-23,3 kcal/mol
596	-31,3 kcal/mol	/
1913	-35 kcal/mol	/
3648	-29,8 kcal/mol	/
3687	-24,6 kcal/mol	/

[a] KrugerJ & Rehmsmeier M, NAR 2006, 24, W451-W454

Table S6

List of primers used thoughout the manuscript

ChIP primers	Forward	Reverse
SREBP2	5′- CCCTCTCCAGCCTCAGACAATGA-3′	5′-GGCACATTCACCTGCTTGCTAACT-3 ′
RAB1A	5′- AACTTGCACTACTGCCTCCTGTCA-3	5′-TTAGGCCACCAGGGATTGTTCACT-3 ′
RAB2B	5′- GCCACTGTGTCTGGCCCATAATTT-3′	5′-CCCTTGATCCTTCTTCCTGTTCCT-3 ′
RAB5B	5′- CTTGTTGGCTGGACATGGT-3 ′	5′-GCTATGGATGAGAGAATGGAGG-3 ′
BECLIN1	5- CAGTACATCCTGAGCCAGAAC-3 ′	5-GGGTGCATGAAGAGGTATAGTC -3 ′
TRAF2	5- CCCAGGTTCAAGTGATTTTCCT-3 ′	5′-CTGGGCATGGTGGCAGATGC-3 ′
STAT1	5- GAGGCAGCCATTCGGGAATCTA-3 ′	5′-CTCACCTCGCAACACCTGATCC-3 ′
LAMA3	5- CACCATGCCTGGCTAAGT-3 ′	5′-TGATATCAAAAGCGCAAGCA-3 ′
DNMT3B	5- CCATAAAGTGACCTTCTGG-3 ′	5′-CTGCTGCTCCTCAATACACCAA-3 ′
hsa-mir-21	5′-CCGGCATAGGTCCATCTCTGCA 3′	5′-TCAGCCCGAAACTTCCCAGAG-3′
hsa-mir-26a-2	5′-CACGTGGCCAGCACTGTTAAATGA-3′	5′-GGTTACTACTGATTTCCCTGCCTG -3′
hsa-mir-26b	5′-AAAGAAGTGCACGGGCAACGG-3′	5′-GCGCTTTCCTCTCCCTCTGTGAA -3′
hsa- mir-129-1	5′-TCTGGGCCTCACTTATGCCAC-3′	5′-ATGCTGGGAGAGCTGTGTGACCTT -3′
hsa-mir-138-2	5′- ACCCTGGTACAAGCCAGAGGAAAT-3′	5′-ACAAGCTGAGGCGGGAAGACATAA-3′
hsa-mir-224	5′-CAGAGCAACCCTGGAAACACTCTT -3′	5′-AGCAGAACAAGATGCCTCTCACGA-3′
hsa-mir-302e	5′-GAAGAGACAACACAGAAGCTCAGCAG-3′	5′-CATGTTAGGTCATTGGGAAGTGAGGG-3′
hsa-mir-551B	5′-CAGAAACTGATGGGACTGATCTGC -3′	5′- TGACGAGATATAGTGGCTTGCCTG-3′
hsa-mir-552	5′-GTGATGGACACACACACACACACA-3′	5′-ACAGCTCACCAGTCTTACCCAGAT -3′
hsa-mir-576	5′- TTGGGTCAAGAGTCAGAAGTTT -3′	5′- TGGCTTCTACTTGTCCTTTCC -3′
hsa-mir-596	5′-GGGAGGAAGGAAGGAAAGAAA -3′	5′-TCTGTGAAAGCTGGATGCTAA -3′
hsa-mir-1913	5′-TTGAAAGTGTTCTGCGCTATCC-3′	5′-ACCTGGGCTGAAGGAGATTTA -3′
hsa-mir-3648	5′-GGCCGTGCCTGAGGTTTCT -3′	5′-AACGGACGTGAAGCCGGTGA -3′

mRNA primers	Forward	Reverse	Probe
SREBP2	5′-GCCCTGGAAGTGACAGAGAG -3′	5′-TGCTTTCCCAGGGAGTGA -3′	#21, cat.no. 04686942001
RAB1A	5′-GGGAAAACAATCAAGCTTCAAA -3	5′-CTGGAGGTGATTGTTCGAAAT -3′	#72, cat. no. 04688953001
RAB2B	5′-ACAAATCAAACTGCAAATCTGG -3′	5′-AACGGGTGATAGAACGGAAG -3′	#61, cat. no. 04688597001
RAB5B	5′-GCCAAGACAGCTATGAACGTG -3 ′	5′-GCCAAGACAGCTATGAACGTG -3′	#72, cat.no. 04688953001
BECLIN1	5'-GCAGCTGGATAAGCTGAAGA-3'	5'-CGACCCAGCCTGAAGTTATT-3'	

miRNAs primers			
miRNAs	***Probe***	***miRNAs***	***Probe***
hsa-mir-21	ID 000397	hsa-mir-452	ID 002329
hsa-mir-26a	ID 000405	hsa-mir-551B -3p	ID 001535
hsa-mir-26b	ID 000407	hsa-mir-551B -5p	ID 002346
hsa-mir-33a	ID 002135	hsa-mir-552	ID 001520
hsa- mir-129-5p	ID 000590	hsa-mir-576-3p	ID 002351
hsa- mir-129-3p	ID 002298	hsa-mir-596	ID 001550
hsa-mir-138	ID 002284	hsa-mir-1913	ID 121113
hsa-mir-224	ID 002099	hsa-mir-3648	ID 464401
hsa-mir-302e	ID 002802	hsa-mir-3687	ID 464645
RNU38B	ID 001004		

**miRNAs PreMir**	
hsa-mir-26a-5p PM10249	hsa-mir-302e PM13467
hsa-mir-138-5p PM11727	hsa-mir-596 PM11608
hsa-mir-224-5p PM12571	

Primers specific for SEED sequences	Forward	Reverse
Seed1	5’-ACCCTGTGACGAACATGGAGAACA-3’	5’-ACACATCCAGCGATAACCAGGACA-3’
Seed2	5’-ACAGGCTTTCACTTTCTCGCCAAC-3’	5’-AGTTCCGATGAGCTTTGCTCCAGA-3’
Seed3	5’-TTTCCATGGCTGCTAGGCTGTACT-3’	5’-AACGTGCAGAGGTGAAGCGAAGT-3’
Seed4	5’-ATAAATTGGTCTGCGCACCAGCAC-3’	5’-ACGGAAGGAAAGAAGTCAGAAGGC-3’
